# Female Dahl, but Not SS13^BN^, Rats Are Susceptible to High-Fat Diet–Induced Hypertension

**DOI:** 10.21203/rs.3.rs-9656583/v1

**Published:** 2026-05-29

**Authors:** Ahmed Elmarakby, Karim Saad, Babak Baban, Hannah Godley-Boswell, Laney Willis, Michael J. Ryan, Jennifer C. Sullivan

**Affiliations:** Augusta University; Augusta University; Augusta University; Augusta University; Augusta University; University of South Carolina; Augusta University

**Keywords:** High fat diet, females, Dahl rats, SS13BN, blood pressure, vascular inflammation

## Abstract

Immune system activation has been implicated in high-fat diet (HFD)-induced elevations in blood pressure (BP) in female Dahl rats. The goal of the current study was to determine the impact of a 10-week HFD on BP, aortic T cell profiles, and vascular function in female Dahl rats and their genetic controls, consomic SS13^BN^ rats. We hypothesized that female Dahl rats would have greater increases in BP, aortic T cell infiltration, and vascular dysfunction in response to a HFD compared to SS13^BN^ rats. Rats were randomized to normal fat diet (NFD) or HFD at 5 weeks of age. Radio-telemeters were implanted at 8 weeks of age and BP was continuously recorded to 15 weeks of age. Aortic T cell profiles were measured by flow cytometry, vascular function by wire myography, and an adipose tissue array was conducted to assess adipokines. While female Dahl rats exhibited HFD-induced hypertension, BP was not different between NFD- and HFD-fed SS13^BN^ controls despite similar caloric intake and increases in body weight in both strains. HFD increased percentages of inflammatory CD4^+^ T cells and T helper 17 cells (Th17) and decreased percentages of anti-inflammatory regulatory T cells (Tregs) in the aorta regardless of rat strain, although SS13^BN^ rats had a less pro-inflammatory immune profile than Dahl rats. Moreover, perivascular adipose tissue (PVAT) attenuated phenylephrine-mediated aortic contraction to a greater extent in SS13^BN^ rats than in Dahl rats fed an HFD. Of the 30 adipokines assessed, visceral adipose tissue expression of the anti-inflammatory adipokines lipocalin-2 and TIMP-1 was reduced in both strains following HFD treatment, with a greater magnitude of decrease in SS13^BN^ rats. Overall, these data indicate that genetic background influences susceptibility to HFD-induced hypertension in females, with enhanced PVAT-mediated regulation of vascular function and a less pro-inflammatory milieu in SS13^BN^ rats.

## Plain English summary

High blood pressure is a major risk factor for heart diseases, and obesity can increase this risk. The reasons why women are more vulnerable to harmful cardiovascular effects of excess body fat are not fully understood. We examined how a high-fat-diet can affect blood pressure, blood vessel function and inflammation in two related strains of female rats: Dahl rats, which are more prone to high blood pressure, and SS13^BN^ rats, which are genetically protected from hypertension during salt intake. Both female strains were fed either a normal or high-fat-diet for 10 weeks. Both strains consumed more calories and gained more weight and fat similarly on the high-fat diet compared to normal diet. However, blood pressure only increased in female Dahl rats, while SS13^BN^ remained protected. The high fat diet increased inflammatory immune cells in aorta in both strains, but Dahl rats had more inflammatory profile. The fat around blood vessels, named perivascular fat, helped to reduce blood vessels contraction more in SS13^BN^ than Dahl rats. Overall, the findings show that genetic background can influence whether a high fat diet can lead to hypertension in females. The results also suggest that hypertension related to obesity does not only depend on body fat or inflammation, but also on how blood vessels and the surrounding fat tissue adapt to dietary stress.

## NEW & NOTEWORTHY

Female Dahl rats provide a powerful model for investigating mechanisms underlying epidemiological evidence that women exhibit greater blood pressure sensitivity to increased adiposity than men. Moreover, identifying mechanisms that protect female SS13^BN^ rats from HFD-induced hypertension, despite comparable vascular T cell infiltration, may reveal novel strategies to preserve BP control and improve cardiovascular health in women.

## Introduction

Hypertension is a leading risk factor for the development of cardiovascular disease (CVD) worldwide and affects half of the adult population in the United States [1]. While the prevalence of hypertension is typically lower in premenopausal women than in age-matched men [2], women represent approximately half of total hypertension cases [3], and CVD remains the leading cause of death in women world-wide. Thus, identifying the biological mechanisms that regulate blood pressure (BP) in females is critically important.

Obesity is classified as an epidemic disease worldwide regardless of age, sex, race and ethnicity, or socioeconomic status [4]. The high obesity rate coincides with the high prevalence of cardiometabolic disease (hypertension, dyslipidemia, insulin resistance, and type II diabetes), all of which increase CVD morbidity and mortality [5]. While premenopausal women generally have a lower prevalence of CVD compared to age-matched men [6], obese women are at higher risk of CVD than age matched obese men [7]. Moreover, recent data from the NHANES indicate that the prevalence of obesity is consistently higher in adult women than in men at all age groups from 20 to 65 years of age [8]. Two-thirds of the hypertension cases in the United States are positively correlated with excessive weight gain [9] and being overweight and obese is a greater risk factor for the development of hypertension in women than men [10]. Thus, understanding why overweight/obese females lose their relative cardiovascular protection vs. age-matched men is necessary to improve the health of women worldwide.

Adiposity promotes chronic low-grade inflammation, which has been linked to the development of hypertension in both sexes [11–15]. Studies show that renal, vascular, and adipose tissue macrophage and T cell infiltration are increased in obesity [16, 17]. Studies also suggest that macrophages and T cells are polarized towards a pro-inflammatory phenotype in the vasculature and adipose tissue of obese animals causing vascular dysfunction and promoting the development of hypertension [14, 18].

Diets rich in saturated fat are commonly used to induce symptoms of human cardio-metabolic disease in rodents [19]. Male and female Dahl rats exhibit increases in BP on a high fat diet (HFD) with normal salt intake [15] and we recently demonstrated a direct role for T cells in HFD-induced increases in BP in male and female Dahl rats [14, 15]. Thus, the current study was designed to increase our understanding of how a HFD increases BP in females. We hypothesized that female Dahl rats will be more susceptible to HFD-induced increases in BP, vascular inflammation, and vascular dysfunction compared to control SS13^BN^ rats. The SS13^BN^ rat model was created by the introgression of chromosome 13 from normotensive Brown Norway rats into the hypertensive Dahl rat background and serves as a genetic control for the Dahl rat in hypertension and related cardiovascular studies [20, 21].

## Methods

### Animals:

All animal experiments were approved by the Augusta University Institutional Animal Care and Use Committee and conducted in accordance with the National Institutes of Health Guide for the Care and Use of Laboratory Animals. Studies included female Dahl rats from a colony maintained at Augusta University originally obtained from the Medical College of Wisconsin (SS/JrHsdMcwi) and SS13^BN^ from Charles River (Wilmington, MA). Rats were maintained in temperature-and humidity-controlled rooms on a 12-hour light:dark cycle with ad libitum access to an AIN purified diet containing 0.4% NaCl (*Dyets, cat# 113755GI*). Rats were weaned at ~21 days of age and maintained on the AIN purified diet. At 5 weeks of age, rats were randomized to receive either a normal fat diet (NFD) with 0.16% kcal from fat (*Bio-serv, cat# F4031*, https://www.bio-serv.com/pdf/F4031.pdf) or a HFD with 59% kcal from fat (major fat component is lard, *Bio-serv, cat # F3282*, https://www.bio-serv.com/pdf/F3282_S3282.pdf) for 10 weeks. Note, both NFD and HFD contain equal amounts of NaCl, 0.4%. Food and water were available ad libitum.

Body weight, food intake, and water intake were measured using metabolic cages every 2 weeks. After 10 weeks of dietary treatment, all rats were euthanized to isolate visceral adipose tissue, spleen and aorta.

### Telemetry BP Measurement.

Following 3 weeks of NFD or HFD treatment, 8-week-old female DSS and SS13^BN^ rats were anesthetized with isoflurane (2% by inhalation) and underwent surgery for implantation of radio-telemetry transmitters (HSD-10, Data Sciences International, St. Paul, MN) into the abdominal aorta (n = 4–5/group). Rats were allowed to recover for one week prior to the initiation of BP measurements. BP was continuously recorded to the end of the 10 weeks of dietary treatment and is reported as 24-hour averages.

### Immunostaining.

Isolated spleen was immediately embedded in optimal cutting temperature (OCT) compound, frozen in liquid nitrogen, and sectioned using a cryostat. Slides were allowed to thaw before being washed with PBS and fixed with 10% formalin. Sections were blocked in 10% horse serum in 0.1% phosphate-buffered saline with Tween (PBST) for 30 minutes. Slides were incubated overnight in primary antibodies to CD3 (Invitrogen, Cat# 14003082) and F4/80 (Abcam, Cat# Ab30042) as markers for T cells and macrophages, respectively, in humidified chamber at 4°C. The next day, slides were washed in 0.1% PBST and incubated with HRP-conjugated donkey anti-mouse IgG (Jackson Immuno Research Laboratories, Cat# 715035020) for 1 hour at room temperature. Slides were then washed, and color was developed with diaminobenzidine (DAB; BioCare Medical, Cat# 5082373). Slides were mounted with cytoseal medium. Images were captured by an investigator blinded to the hypothesis utilizing bright-field settings at 200X magnification on a digital camera (Olympus DP12; Olympus America). The number of CD3^+^ and F4/80^+^ cells were counted per slide, and the average number was reported per 0.15 mm^2^ slide area.

#### Proteome Profiler Array

Visceral adipose tissue (VAT) isolated from female SS13^BN^ and Dahl rats following NFD or HFD treatment was used for proteomic profiling using a rat adipokine array (R&D Systems; Cat. #ARY016). Briefly, VAT was homogenized in PBS with protease inhibitors then Triton X100 was added. Samples were centrifugated at 10,000 × g for 5 minutes to remove cellular debris and protein concentration was quantified using Pierce BCA Protein Assay Kit (Thermo-Fisher, Cat# 23223). Samples were pooled by group, and equal amounts of total protein (approximately 400 μg per group) were used for adipokine array analysis according to the manufacturer’s instructions. Fold changes in adipokine expression following HFD treatment were calculated relative to NFD within each rat strain and used to generate a heat map using GraphPad.

### Vascular function and T cell assessments.

In a separate set of female Dahl and SS13^BN^ rats randomized to a NFD or HFD for 10 weeks (n = 5–6/group), thoracic aorta was isolated to measure vascular reactivity and aortic T cell profiles using wire myograph and flow cytometry, respectively.

#### Vascular Reactivity.

Half of the thoracic aorta was used to assess vascular function. Since PVAT has been shown to regulate vascular function [22, 23] and the PVAT is a rich source of immune cells [24], sections of aorta were studied with PVAT intact or removed. Aorta were cut into 2 mm rings and mounted on pins for isometric myography (Danish Myo Technology A/S, Aarhus, Denmark) in chambers filled with aerated (95% O2 and 5% CO2) Krebs buffer (130 mM NaCl, 4.7 mM KCl, 1.17 mM MgSO_4_, 14.9 NaHCO_3_, 5.6 mM Dextrose, 1.56 mM CaCl_2_H_2_O, 0.03 mM EDTA) heated to 37°C. The tension was adjusted to 30 mN and rings were allowed to equilibrate for 30 minutes with the Krebs buffer replaced every 15 minutes before the viability of the vessel was determined by a robust vasoconstrictor response to 10^− 6^ M phenylephrine (PE) followed by vasorelaxation to 10^− 6^ M acetylcholine (ACh). Only arteries that relaxed at least 80% of the maximal PE-induced contraction were included in the study. Vasoconstriction or vasodilation was assessed by performing cumulative concentration response (CCR) curves to PE (10^− 10^-10^− 4^ M) or to the endothelium-dependent dilator ACh (10^− 10^-10^− 4^ M) after vessel pre-constricted with PE (10^− 6^). Vasoconstriction data were presented as % contraction from baseline values. Vasorelaxation data were presented as % relaxation from PE constriction

#### Flow Cytometry.

The remaining half of the thoracic aorta was isolated for flow cytometric analysis with intact PVAT as previously described [14]. Briefly, the thoracic aorta was flushed with phosphate-buffered saline (PBS) to clear residual blood following isolation, minced into pieces and digested in RPMI plus 10% FBS containing collagenases A and B at 37°C for 30 minutes to generate a single cell suspension. The single cell suspension was then passed through a 70-micron strainer in PBS and centrifuged at 1400 rpm for 10 minutes. Cells were lysed with FACS Lysing Buffer (BD Biosciences), washed with PBS, and centrifuged [15]. Single cell suspensions were stained with antibodies for T cell surface markers CD3 (1:100, eBioscience) and CD4 (1: 100, BD Biosciences) for 15 minutes on ice in the dark. Cells were then washed with PBS, fixed and permeabilized using fix/perm concentrate (eBioscience) before incubation with antibodies for intracellular staining for FOXP3 (1:100, eBioscience) to identify Tregs (CD3^+^ CD4^+^FOXP3^+^) or RORγt (1:100, R&D Systems) to identify Th17 cells (CD3^+^ CD4^+^ RORγt^+^). Cells were then washed and run through a four-color flow cytometer (FACS Calibur, BD Biosciences) and data were collected using Cell Quest. Compensation and gating were performed using FlowJo.

### Statistical Analysis.

All data are presented as mean ± SEM. Flow cytometry and metabolic data were compared using two-way ANOVA followed by Tukey’s post-hoc test. Body weight, BP and vascular reactivity data were analyzed using repeated-measures ANOVA and between group comparisons were made using a 2-way ANOVA followed by Tukey’s post-hoc test. Analyses were performed using GraphPad Prism Version 8.0 software (GraphPad Software Inc, La Jolla, CA) and for all comparisons, differences were considered statistically significant with P < 0.05.

## Results

### HFD increases total fat and caloric intake independent of body weight changes compared to NFD.

**Figure 1:**
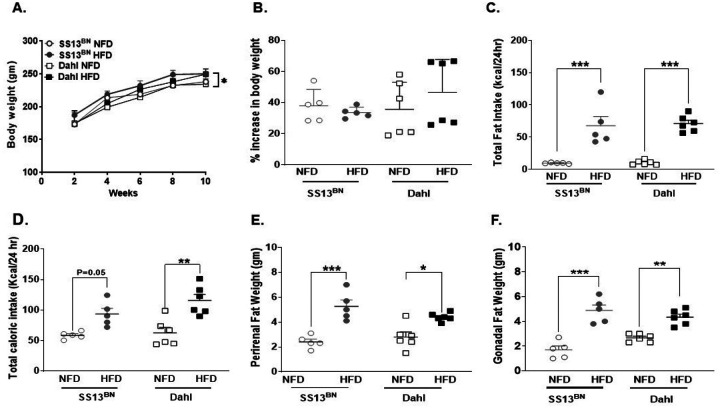
HFD increases total fat and caloric intake and perirenal and gonadal fat deposition independent of body weight changes compared to NFD. Female SS13^BN^ and Dahl rats were randomized to a NFD or HFD from 5 to 15 weeks of age; n=5–6. Body weight was measured biweekly (A). Percent increase in body weight from week 2 to week 10 of diet treatment (B), fat (C) and caloric intake (D) were calculated based on 24-hour metabolic cage data at the end of the diet treatment. At this time, peri-renal (E) and gonadal (F) adipose tissue were isolated and weighed. * Indicates p<0.05, ** indicates p<0.005 and *** indicates p<0.0005.

There was an age-related increase in body weight in female Dahl and SS13^BN^ controls from week 2 to week 10 of treatment regardless of diet (Fig. 1A; P < 0.05). The percent increase in body weight in response to the HFD or NFD from week 2 to week 10 of treatment was also calculated (Fig. 1B). HFD did not result in greater percent increases in body weight compared to NFD in either strain (P_diet_=0.97; P_strain_=0.77; P_interaction_= 0.53). However, total fat intake was significantly greater in rats on a HFD compared to a NFD regardless of strain (P_diet_<0.0001; P_strain=_0.77; P_interaction_=0.85; Fig. 1C). Similarly, HFD fed rats consumed more total calories compared to rats on a NFD regardless of strain (P_diet_<0.0001; P_strain_=0.14; P_interaction_=0.30 Fig. 1D). Perirenal (P_diet_: p < 0.0001; P_strain_= 0.60; Fig. 1E) and gonadal fat pad weights (P_diet_<0.0001; P_strain_= 0.57; Fig. 1F) were also greater in HFD fed rats regardless of strain, and the increase tended to be greater in female SS13^BN^ vs. Dahl rats (P_interaction_=0.08 and P_interaction=_0.03, respectively).

### Female Dahl rats are susceptible to HFD-induced hypertension; SS13 ^BN^ controls are resistant to HFD-induced increases in BP.

**Figure 2:**
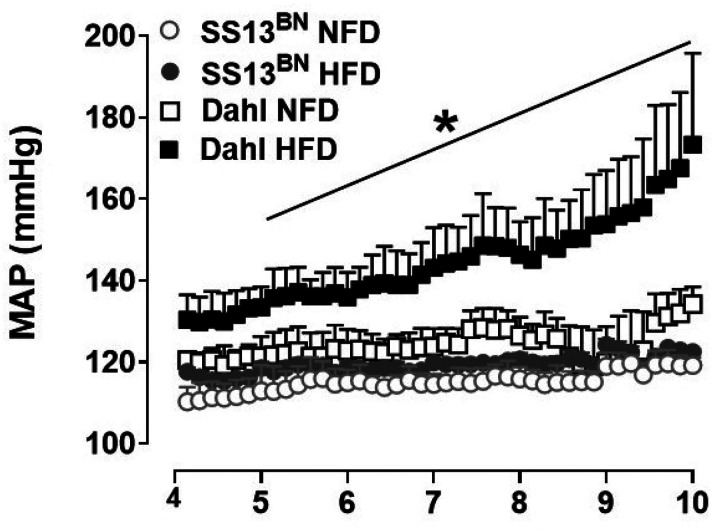
Female Dahl rats are susceptible to HFD-induced hypertension while SS13^BN^ controls are resistant. Female SS13^BN^ and Dahl rats were randomized to a NFD or HFD from 5 to 15 weeks of age; n=4–5. Radio telemeters were implanted 3 weeks after the initiation of the dietary treatments. * Indicates p<0.05 vs. all other groups.

The impact of HFD vs. NFD on BP in female Dahl and SS13^BN^ rats was measured by radiotelemetry (Fig. 2). HFD resulted in a significantly greater age-related increase in BP in female Dahl rats compared to female Dahl rats on a NFD or female SS13^BN^ on a NFD or HFD. This effect was significant by the 5th week of HFD treatment and BP remained higher in Dahl rats throughout the remainder of the diet treatment (P < 0.05). In contrast, there were no differences in BP in female SS13^BN^ on a NFD vs. HFD.

### HFD increases splenic macrophage and T cell counts with higher levels in Dahl vs. SS13 ^BN^ rats.

**Figure 3:**
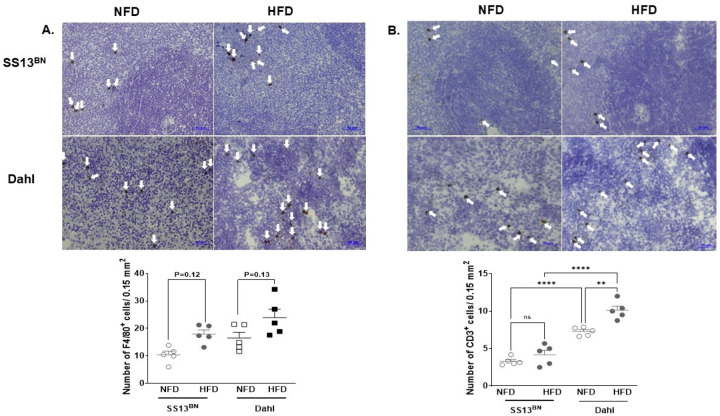
HFD increases splenic macrophage and T cell counts with greater levels in Dahl vs. SS13BN rats. Female SS13^BN^ and Dahl rats were randomized to a NFD or HFD from 5 to 15 weeks of age; n=5. Following 10 weeks of dietary treatment, spleen were isolated, snap-frozen, and sectioned using a cryostat. Sections were used for immunohistochemical assessment of F4/80^+^ macrophages (A) and CD3^+^ T cells (B). Representative images at 200X magnification and average numbers of F4/80^+^ cell and CD3^+^ cell were reported per 0.15mm^2^ cross sectional area. ** Indicates p<0.005 and *** indicates p<0.0005.

Splenic F4/80^+^ and CD3^+^ cells were quantified as markers of macrophages and T cells, respectively, in Dahl and SS13^BN^ rats following NFD and HFD treatment. Numbers of F4/80^+^ cells were higher in Dahl rats vs. SS13^BN^ rats and higher in rats on HFD vs NFD (P_diet_=0.003; P_strain=_0.01; P_interaction_=0.98, Fig. 3A). Similarly, numbers of CD3^+^ cells were greater in Dahl vs. SS13^BN^ control rats on NFD. HFD significantly increased CD3^+^ cells only in Dahl rats (P_diet_=0.007; P_strain_<0.0001; P_interaction_ =0.05, Fig. 3B).

### HFD increases aortic T cell infiltration in female rats; Dahl rats have a more pro-inflammatory T cell profile than SS13 ^BN^ rats.

**Figure 4:**
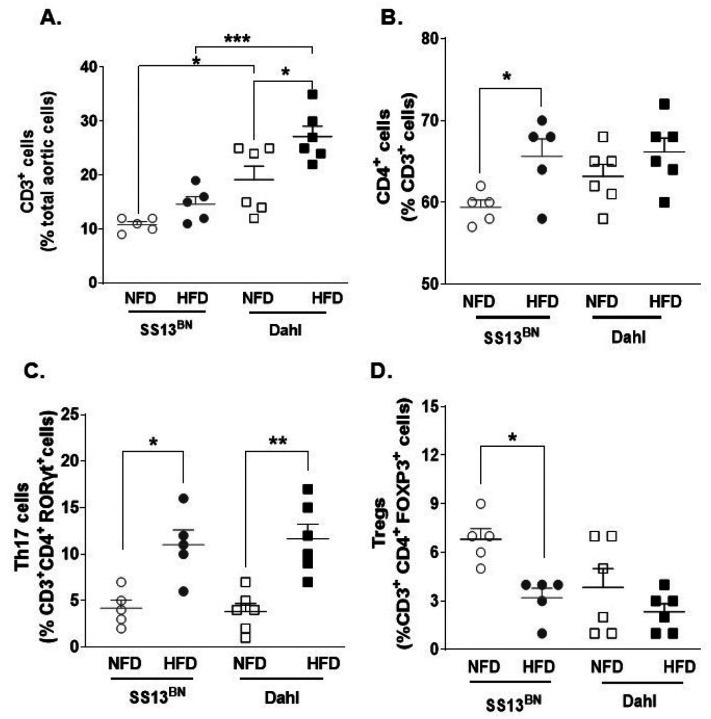
HFD increases aortic T cell infiltration in female rats; Dahl rats have a more pro-inflammatory T cell profile than SS13^BN^ rats. Female SS13^BN^ and Dahl rats were randomized to a NFD or HFD from 5 to 15 weeks of age; n=5–6. Following 10 weeks of dietary treatment, the aortic T cell profile was measured by flow cytometry. The percentages show are the total CD3^+^ T cells (% of total aortic cells, A), CD4^+^ T cells (% of CD3^+^ cells, B), Th17 cells (% CD3^+^ CD4^+^ RORγt^+^, C) and Tregs (% CD3^+^ CD4^+^ FOXP3^+^ cells, D). * Indicates p<0.05, ** indicates p<0.005 and *** indicates p<0.0005.

To determine if HFD-induced increases in BP are accompanied by strainspecific increases in vascular T cell infiltration, the aortic T cell profile was measured by flow cytometry. The percentage of total aortic CD3^+^ T cells was higher in HFD fed rats (P_diet_=0.006), although female Dahl rats had more total T cells than SS13BN controls regardless of diet (Pstrain<0.0001; Pinteraction=0.28; Fig. 4A). The percentage of CD4^+^ T cells and pro-inflammatory CD3^+^CD4^+^Th17 cells were also greater in aorta from HFD compared to NFD fed rats (Fig. 4B: CD4: P_diet_=0.01; P_strain=_19; P_interaction_: p = 0.33; Fig. 4C: Th17: P_diet_<0.0001; P_strain_=0.54; P_interaction_=0.74). Although aorta from SS13^BN^ female controls had a higher percentage of the anti-inflammatory CD3^+^CD4^+^Tregs than Dahl rats (P_strain_=0.03; Fig. 4D), HFD resulted in comparable decreases in Tregs vs. same strain of NFD fed rats (P_diet_=0.005; P_interaction_=0.21; Fig. 4D).

### Vascular function is not altered in female rats fed a HFD in the absence of PVAT.

**Figure 5:**
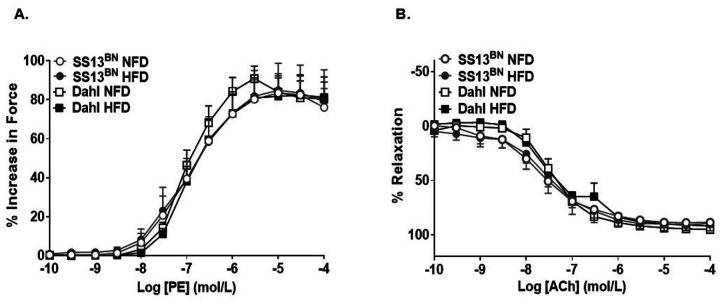
Vascular function is not altered in female rats fed a HFD in the absence of PVAT. Female SS13^BN^ and Dahl rats were randomized to a NFD or HFD from 5 to 15 weeks of age; n=5–6. Following 10 weeks of dietary treatment, aortic rings were isolated and cleaned of adherent fat. Aortic vasoconstrictor responses to phenylephrine (PE, A) or endothelial dependent relaxation to acetylcholine (ACh, B) were measured by wire myography.

Aortic rings, cleaned of PVAT, were assessed for contractile and relaxation responses to increasing concentrations of PE and ACh, respectively. PE induced comparable concentration-dependent contraction in aorta isolated from all rat groups regardless of diet (Fig. 5A). Similarly, ACh-induced concentration dependent relaxation in aortic rings was similar in female SS13^BN^ and Dahl rats on NFD, and HFD did not alter this response (Fig. 5B).

### PVAT buffers aortic contraction on HFD, and the effect is greater in SS13 ^BN vs.^ Dahl rats.

**Figure 6:**
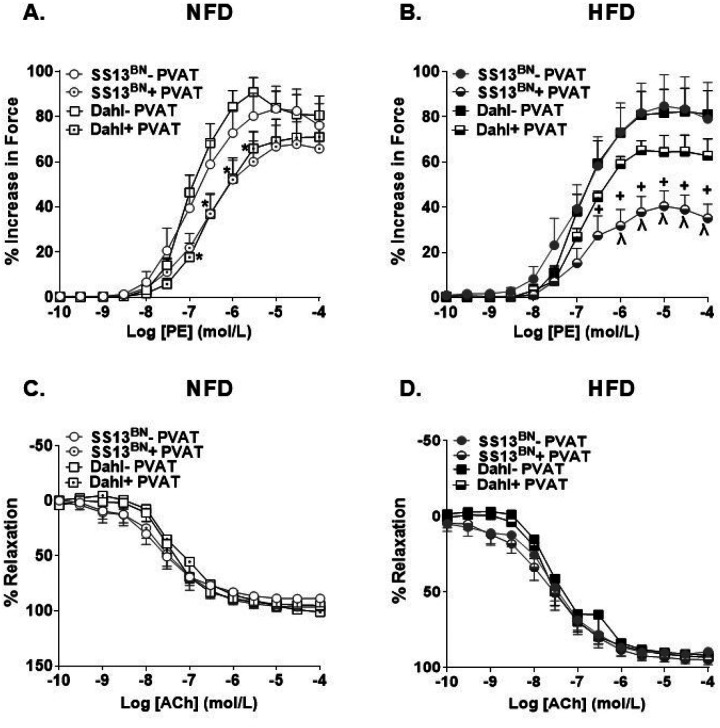
PVAT buffers the aortic contractile response to PE without impacting endothelial dependent relaxation to ACh in female SS13^BN^ rats on a HFD. SS13^BN^ and Dahl rats were randomized to a NFD or HFD from 5 to 15 weeks of age; n=5–6. Following 10 weeks of dietary treatment, aortic rings were isolated and PVAT was removed or left intact. Aortic vasoconstrictor responses to PE or endothelial dependent relaxation to ACh were measured by wire myography in both rat strains following NFD (A & C) or HFD (B & D) treatment. * Indicates p<0.05 vs. corresponding values in absence of PVAT following a NFD treatment. ^+^ indicates p<0.05 vs. corresponding values in absence of PVAT following a HFD treatment. ^λ^indicates p<0.05 vs. corresponding values in presence of PVAT in female Dahl rats following a HFD treatment.

When PVAT remained intact on aortic segments following treatment with a NFD, PE-induced contraction was blunted in both female SS13^BN^ and Dahl rats vs. aortic segments without PVAT, although this only reached significance in Dahl rats (P < 0.05, Fig. 6A). In contrast, PVAT resulted in a significantly greater attenuation of contraction in aortic segments from SS13^BN^ rats compared to Dahl rats on HFD. PVAT mediates a 55.6 ± 7.9% decrease in maximal contraction to PE in SS13^BN^ vs a 22.7% ± 9.1% decrease in Dahl rats (P < 0.026, Fig. 6B). Aortic relaxation in response to increasing concentration of ACh was not altered by the presence of PVAT in either SS13^BN^ or Dahl rat on either a NFD (Fig. 6C) or a HFD (Fig. 6D).

### Female SS13BN rats exhibit greater expression of anti-inflammatory adipokines in VAT than Dahl rats; HFD reduced adipokine expression in both strains, with a larger reduction in SS13BN rats.

**Figure 7:**
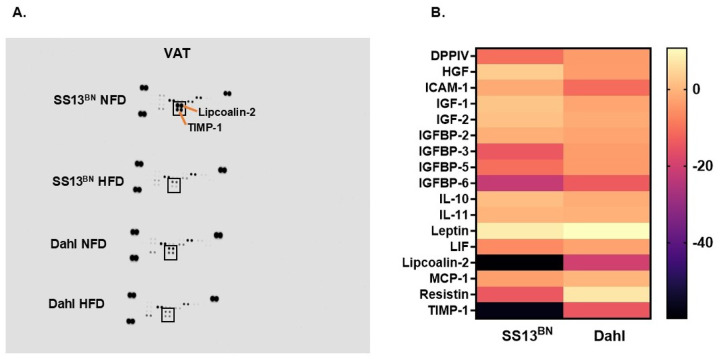
Female SS13^BN^ rats showed greater VAT anti-inflammatory adipokine expression than Dahl rats; HFD treatment reduced their levels in both strains, with a larger decrease in SS13^BN^ rats. VAT isolated from female SS13^BN^ and Dahl rats following NFD or HFD treatment was used for proteomic profiling using a rat adipokine array, with adipokine expression reported as percent change in HFD relative to NFD within each strain (B); n=5. Expression levels of 17 adipokines were detected in both rat strains during NFD and HFD treatment (A). The percent changes in expression were used to create a heat map using GraphPad software (B).

VAT isolated from female SS13^BN^ and Dahl rats following NFD or HFD treatment was used for proteomic profiling using a rat adipokine array, with adipokine expression reported as percent change in HFD relative to NFD within each strain. Out of 30 adipokines, 17 were detected in VAT isolated from both rat strains after NFD and HFD treatment. Interestingly, the most pronounced changes were in expression levels of the anti-inflammatory adipokines lipocalin-2 and TIMP-1. For both, expression was greater in SS13^BN^ rats vs. Dahl following NFD (Fig. 7). Although HFD treatment decreased VAT expression levels of both lipocalin-2 and TIMP-1 in SS13^BN^ and Dahl rats, the decrease was greater in SS13^BN^ vs. Dahl rats (Fig. 7).

## Discussion

Based on epidemiological evidence indicating that women have greater susceptibility than men to the negative cardiovascular consequences of increases in adiposity [7, 25], there is a need to better understand how a HFD predisposes females to increases in BP. In the current study, we found that despite a HFD causing comparable increases in body weight and greater increases in fat deposition in female SS13^BN^ rats compared to Dahl rats, only Dahl rats exhibited an increase in BP. While HFD was associated with increases in the pro-inflammatory T cell status in both strains, Dahl rats had a higher percentage of aortic CD3^+^ T cells and fewer anti-inflammatory Tregs than SS13^BN^, and PVAT buffered vascular contraction to a greater extent following a HFD in female SS13^BN^ rats. Understanding the mechanisms driving greater increases in inflammation and BP in female Dahl rats and protecting female SS13^BN^ rats from HFD-induced increases in BP may provide novel insights to improve the cardiovascular health of females.

We previously showed that female Dahl rats maintained on a HFD for either 4 weeks [15] or 10 weeks [14] exhibit similar increases in systolic BP to their male counterparts, demonstrating that female Dahl rats are susceptible to HFD-induced hypertension. This is consistent with the work of others [12, 22] and confirmed in the present study. In contrast, female SS13^BN^ controls are resistant to HFD-induced hypertension, similar to what has previously been shown in male SS13^BN^ [26, 27]. Our findings further support the use of the Dahl rat model to better understand the mechanisms driving increases in CVD on a chronic HFD in females.

Macrophages and T cells play a role in the incidence and progression of cardiometabolic disease [28]. Increased renal macrophage and T cell infiltration are highly correlated with elevation sin BP and renal inflammation in male Dahl rats after HFD treatment [12], and T cells in particular have been implicated in the pathogenesis of HFD-induced hypertension in male Dahl rats [15, 26, 29]. Additionally, we previously showed that four weeks of HFD promotes vascular T cell infiltration and a pro-inflammatory T cell profile in female Dahl treated with HFD from 12 to 16 weeks of age [15], and we recently published that male and female Dahl rats lacking T cells have an attenuated increase in BP on a HFD vs. wild-type control Dahl rats [14]. In the current study, female Dahl rats had greater increases in total T cell infiltration than SS13BN rats and were more susceptible to HFD-induced hypertension.

The SS13^BN^ rat was generated by the introgression of chromosome 13 from the Brown Norway rat into the Dahl genetic background [20, 21]. This resulted in the attenuation of salt-sensitive hypertension in the consomic strain, making SS13^BN^ an excellent genetic control for diet-induced hypertension in the Dahl rat [21, 26, 27]. The absence of a BP response to a HFD in female SS13^BN^ rats vs. female Dahl rats supports the importance of genes located on chromosome 13 in BP regulation during HFD treatment. Importantly, HFD induced aortic inflammation in female SS13^BN^ rats without a corresponding increase in BP. The dissociation between vascular inflammation and BP elevation in SS13^BN^ rats suggests additional protective mechanisms, but these are not directly tested in the current study. Alternatively, the contribution of additional chromosomes to HFD-induced increases in BP and inflammation can be examined. Viel et al. previously showed that chromosome 2 plays a role in regulating vascular inflammation [33] since male SS^BN2^ rats, in which chromosome 2 from the Brown Norway rat was transferred to the Dahl rat, exhibit reduced aortic CD4^+^ T cell infiltration and blunted expression of inflammatory markers and mediators, including NFκB, CCR5, VCAM-1, and ICAM-1 compared to Dahl rats when maintained on a normal salt (0.23% NaCl) diet [33].

PVAT plays a role in regulating vascular function, especially in obesity when more fat is deposited around vessels (19, 28). PVAT enhances the ability of arteries to relax in response to stretch and is known to have an anticontractile effect, reducing the maximum contraction to PE [22, 23]. Consistent with this, vascular contractility to PE was attenuated by PVAT in isolated aortic rings from SS13^BN^ and Dahl rats on a HFD, yet the impact was greater in the SS13^BN^ rats- suggesting that alterations in PVAT function contribute to the strain difference in BP response to a HFD. The current findings are similar to work from Watts et al. who reported that PE-induced contraction was not significantly altered by PVAT in female Dahl rats fed a HFD [22]. Consistent with previous studies [22], our data demonstrate that aortic endothelial function is similar in female Dahl and SS13^BN^ rats, and PVAT did not alter ACh-mediated relaxation in either female SS13^BN^ or Dahl rats following NFD or HFD treatment.

Studies have shown that VAT is metabolically active, as HFD promotes adipocyte expansion and immune cell infiltration [13]. Previous work has identified lipocalin-2 and TIMP-1 as adipokines with important roles in metabolic inflammation and tissue remodeling. Lipocalin-2, produced by both adipocytes and macrophages, can be released into the circulation during HFD exposure [35]. TIMP-1 is a key modulator of extracellular matrix turnover, counterbalancing matrix metalloproteinase activity and influencing fibrosis and structural remodeling in adipose tissue during obesity [36]. Changes in tissue levels of these factors during HFD may therefore reflect adaptive remodeling processes rather than simple suppression of anti-inflammatory pathways. Indeed, prior studies indicate that HFD is associated with increased matrix remodeling and altered adipokine trafficking [11], which may result in reduced tissue levels despite heightened systemic inflammatory signaling. We suggest that observed decreases in lipocalin-2 and TIMP-1 in VAT after HFD could be attributed to enhanced systemic secretion and extracellular matrix remodeling rather than reduction in their overall production. The greater reductions in SS13^BN^ rats vs. Dahl after HFD treatment also suggests a more pronounced adaptive response to dietary challenge in SS13^BN^.

In conclusion, our findings support the concept that genetic background shapes how adipose tissue and the vasculature respond to dietary excess. While obesity-related inflammation is commonly associated with hypertension, emerging evidence suggests that genetic and physiological context, including adaptive capacity, modulates susceptibility independent of adiposity or immune activation alone. Given that PVAT is a rich source of immune cells, differences in PVAT-mediated vascular regulation may contribute to strain-specific susceptibility to HFD-induced hypertension. Further investigation into the mechanisms conferring protection in SS13^BN^ rats may provide insight into factors that preserve BP regulation in females in the setting of increased adiposity.

## Data Availability

The data underlying this study are available upon reasonable request from the corresponding author.

## Highlights

- Female Dahl rats, but not SS13^BN^ rats were sensitive to high fat diet-induced hypertension despite comparable increases in caloric intake and adiposity.
- High-fat diet increased aortic inflammatory T cell profile in both rat strains; however, Dahl rats showed a more aortic pro-inflammatory immune profile than SS13^BN^.
- SS13^BN^ rats were protected from blood pressure elevation during high fat diet treatment despite high fat diet-induced vascular inflammation, suggesting that increased vascular inflammation alone might not be the sole mechanism for susceptibility to hypertension.
- Perivascular adipose tissue buffered aortic contraction in SS13^BN^ rats on high-fat diet, indicating a possible vascular protective mechanism that limits their susceptibility to hypertension.
